# Enzymatic synthesis of chiral amino‐alcohols by coupling transketolase and transaminase‐catalyzed reactions in a cascading continuous‐flow microreactor system

**DOI:** 10.1002/bit.26470

**Published:** 2017-11-09

**Authors:** Pia Gruber, Filipe Carvalho, Marco P. C. Marques, Brian O'Sullivan, Fabiana Subrizi, Dragana Dobrijevic, John Ward, Helen C. Hailes, Pedro Fernandes, Roland Wohlgemuth, Frank Baganz, Nicolas Szita

**Affiliations:** ^1^ Department of Biochemical Engineering University College London London United Kingdom; ^2^ Department of Bioengineering and IBB—Institute for Bioengineering and Biosciences Instituto Superior Técnico Universidade de Lisboa Lisboa Portugal; ^3^ Department of Chemistry University College London London United Kingdom; ^4^ Faculty of Engineering Universidade Lusófona de Humanidades e Tecnologias Lisboa Portugal; ^5^ Sigma–Aldrich Member of Merck Group Buchs Switzerland

**Keywords:** cascading reactor system, continuous‐flow microreactors, multi‐step bioconversion, transaminase, transketolase

## Abstract

Rapid biocatalytic process development and intensification continues to be challenging with currently available methods. Chiral amino‐alcohols are of particular interest as they represent key industrial synthons for the production of complex molecules and optically pure pharmaceuticals. (2*S*,3*R*)‐2‐amino‐1,3,4‐butanetriol (ABT), a building block for the synthesis of protease inhibitors and detoxifying agents, can be synthesized from simple, non‐chiral starting materials, by coupling a transketolase‐ and a transaminase‐catalyzed reaction. However, until today, full conversion has not been shown and, typically, long reaction times are reported, making process modifications and improvement challenging. In this contribution, we present a novel microreactor‐based approach based on free enzymes, and we report for the first time full conversion of ABT in a coupled enzyme cascade for both batch and continuous‐flow systems. Using the compartmentalization of the reactions afforded by the microreactor cascade, we overcame inhibitory effects, increased the activity per unit volume, and optimized individual reaction conditions. The transketolase‐catalyzed reaction was completed in under 10 min with a volumetric activity of 3.25 U ml^−1^. Following optimization of the transaminase‐catalyzed reaction, a volumetric activity of 10.8 U ml^−1^ was attained which led to full conversion of the coupled reaction in 2 hr. The presented approach illustrates how continuous‐flow microreactors can be applied for the design and optimization of biocatalytic processes.

## 1 INTRODUCTION

Expeditious and stereoselective synthetic methodologies for chiral amino‐alcohols from simple starting materials are of significant interest (Shi, Wong, & Buchwald, 2016; Weinstein, Schuman, Tan, & Stahl, 2013). Chiral amino‐alcohol moieties occur naturally in a large number of biologically active compounds and are synthesized for various applications such as new‐to‐nature pharmaceuticals, key industrial synthons for the production of complex molecules and optically pure pharmaceuticals, that is, HIV protease inhibitors (Kaldor et al., 1997; Kwon & Ko, 2002) or ephedrine‐type structures produced via multi‐step reactions as recently described by Sehl, Maugeri, and Rother (2015a).

Chiral amino‐triols in particular represent important structural elements in sphingosine‐like metabolites such as myriocin, which inhibits serine palmitoyl transferase and thereby ceramide and sphingolipid biosynthesis (Miyake, Kozutsumi, Nakamura, Fujita, & Kawasaki, 1995). Myriocin was discovered to have antifungal activity (Kluepfel et al., 1972), strong immunosuppressant activity and provided a scaffold for the development of the synthetic analogue FTY720, leading to fingolimod as the first orally applicable treatment of multiple sclerosis (Chun & Brinkmann, 2011). It has been estimated that up to 50% of all pharmaceuticals contain a chiral amine in their structure (Ghislieri & Turner, 2014; Zhu & Hua, 2009). In addition, chiral amino‐alcohols represent useful building blocks for the preparation of chiral catalysts, ligands, and auxiliaries in asymmetric synthesis (Birrell & Jacobsen, 2013).

Chemical routes for the production of these compounds typically require the use of protecting groups, expensive transition metal catalysts and multiple steps for intermediate and final product recovery and purification (Ghislieri & Turner, 2014; Tamura, Tamura, Takeda, Nakagawa, & Tomishige, 2014). In contrast, biocatalytic routes for the synthesis of chiral amino‐alcohols offer an attractive and robust alternative due to their environmentally benign nature and high selectivity (Höhne & Bornscheuer, 2009; Ward & Wohlgemuth, 2010; Wohlgemuth, 2010). Enzymes for the synthesis of chiral amino‐alcohols include imine reductases (Leipold, Hussain, Ghislieri, & Turner, 2013), amino acid dehydrogenases (Zhu & Hua, 2009), transaminases, lyases, and monoamine oxidases (Ghislieri & Turner, 2014; Höhne & Bornscheuer, 2009). Although transaminases with high substrate regio‐ and stereoselectivity have been found, their application is often constrained by unfavorable reaction equilibria and substrate and/or product inhibition (Rios‐Solis et al., 2015; Stewart, 2001; Taylor, Pantaleone, Senkpeil, & Fotheringham, 1998; Villegas‐Torres et al., 2015). These constraints require careful consideration in biocatalytic process development. This becomes an even more demanding task where more than one enzymatic reaction step is required in the synthesis of the chiral amino‐alcohols.

Performing organic synthesis in structured microreactors offers a number of advantages, including increased control over the reaction conditions (i.e., reaction time, flow rate, and reagent addition), reduced use of resources, faster mass and heat transport due to the high surface‐to‐volume ratios and shortened diffusion path lengths. In the specific case of multi‐enzyme reactions, microreactors offer straightforward and efficient compartmentalization of the reactions (Gruber, Marques, O'Sullivan et al., 2017). Furthermore, reaction and separation steps can be combined (O'Sullivan et al., 2012) and throughput can be increased via parallelization of the microreactors (Bolivar, Wiesbauer, & Nidetzky, 2011; Krühne et al., 2014; Wohlgemuth, Plazl, Žnidaršič‐Plazl, Gernaey, & Woodley, 2015). These advantages may lead to faster biocatalytic process development or the creation of novel synthetic routes, while at the same time reducing cost and environmental impact.

**Scheme 1:**
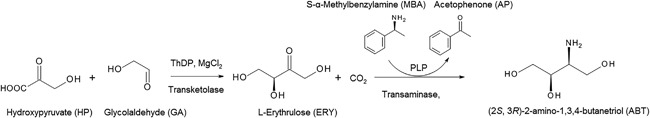
Reaction scheme for the two‐step transketolase‐transaminase catalyzed synthesis of (2*S*,3*R*)‐2‐amino‐1, 3, 4‐butanetriol. PLP, pyridoxal‐5′‐phosphate; ThDP, thiamine diphosphate, and HPA from Lithium‐β‐hydroxypyruvate

In this contribution, we investigate a novel approach for the 2‐step‐enzymatic synthesis of the chiral amino‐triol (2*S*,3*R*)‐2‐amino‐1,3,4‐butanetriol (ABT) based on coupled microreactors. ABT is used as a building block for statins employed in the synthesis of the HIV‐protease inhibitor Nelfinavir (Kaldor et al., 1997; Kwon & Ko, 2002) and as an intermediate in the synthesis of detoxinine, a detoxifying agent for reducing the toxicity of the antibiotic treatment for rice blast disease (Delle Monache et al., 1999; Ingram et al., 2007). In this work, ABT is obtained by coupling a transketolase (TK)‐catalyzed asymmetric carbon‐carbon bond formation with a transaminase (TAm)‐catalyzed conversion of the keto‐group into a chiral amino group (Scheme 1) (Ingram et al., 2007; Rios‐Solis et al., 2011; Villegas‐Torres et al., 2015). We show, for the first time, a microreactor‐based reaction cascade with free enzymes and we obtained for the first time full conversion of this reaction sequence in both continuous‐flow microreactors and in batch. Halim, Rios‐Solis, Micheletti, Ward, and Lye (2014) also achieved full conversion in microtiter plates for the TAm‐catalyzed reaction, however, needed a secondary reaction to drive the reaction equilibrium, and used purified erythrulose (ERY) as a starting material, thus did not perform a coupled reaction. Furthermore, the compartmentalization afforded by our microreactor cascade led to the discovery that a cofactor of the TK reaction inhibits the TAm, which was not previously reported. These advances reinforce the potential of coupled microreactors for biocatalytic process development (Gruber, Marques, O'Sullivan et al., 2017).

## 2 MATERIALS AND METHODS

Unless otherwise stated, all chemicals and reagents were purchased from Sigma–Aldrich (Gillingham, UK) and were used without further purification.

### 2.1 Analytical methods

#### 2.1.1 Protein quantification

Bradford reagent (Sigma–Aldrich) was used for total protein concentration quantification with bovine serum albumin (BSA, Sigma–Aldrich) as standard (Bradford, 1976).

#### 2.1.2 Enzyme activity

##### Transketolase activity measurement

Quantification was carried out in 96‐well microtiter plates by adding 150 μl of transketolase (TK) lysate solution (15 μl of TK lysate, 2.4 mM thiamine diphosphate, ThDP, and 9.8 mM MgCl_2_) to 150 μl of a solution of 100 mM hydroxypyruvate (HPA) and 100 mM glycolaldehyde (GA). Both solutions were prepared in 50 mM Tris–HCl pH 7.0 and incubated at 20°C for 30 min. Using a sacrificial well approach, samples of 50 μl were removed each minute for 5 min and quenched with 450 μl 0.1% (v/v) trifluoroacetic acid (TFA), centrifuged (5,000 rpm, 5 min) and analyzed by HPLC. One transketolase unit (U) was defined as the amount of transketolase which catalyzed the conversion of 1 μmol of substrate per minute at pH 7.0 and 20°C.

##### Transaminase activity measurement

Quantification was carried out in 96‐well microtiter plates by adding 20 μl of transaminase (TAm) (1:10 diluted) solution containing 1 mM pyridoxal‐5′‐phosphate (PLP) to a 180 μl solution of 10 mM (S)‐α‐methylbenzylamine (MBA) and 10 mM sodium pyruvate (PYR). Both solutions were prepared in 50 mM Tris–HCl pH 7.4 and incubated at 20°C for 30 min. Acetophenone (AP) formation was recorded for 2 min in 20 s interval using a plate reader (FLUOstar OPTIMA, BMG Labtech, Ortenberg, Germany) at 280 nm. For activity determination of TAm extracted into pH 9 Tris–HCl buffer, all solutions were prepared at pH 9 accordingly. One transaminase unit (U) was defined as the amount of transaminase that catalyzed the formation of 1 μmol of product (AP) per minute at pH 7.4 and pH 9, respectively, and 20°C.

#### 2.1.3 Reagent analysis by HPLC

HPA and ERY were analyzed directly using an Aminex HPX‐87H column (300 mm × 7.8 mm; Bio‐Rad, UK) with 0.6 ml min^−1^ isocratic elution of 0.1% (v/v) TFA at 60°C and detection at 210 nm. MBA and AP were quantified with an ACE 5 C18 RP column (150 mm × 4.6 mm, 5 μm particle size, Advance Chromatography Technologies, UK). The mobile phase was comprised of 0.1% (v/v) TFA at 1.0 ml min^−1^ with a gradient of acetonitrile from 15% to 72% over 9 min, followed by a 2 min equilibration and detection at 254 nm. 2‐Amino‐1,3,4‐butanetriol (ABT) was derivatized by diluting the samples with 0.2 M borate buffer pH 8.8 and adding an excess of 6‐aminoquinolyl‐*N*‐hydroxysuccinimidyl carbamate. ABT was quantified using an ACE 5 C18 RP column (150 mm × 4.6 mm, 5 μm, Advanced Chromatography Technologies) with a mobile phase comprised of 140 mM sodium acetate buffer (adjusted to pH 5 using acetic acid) at 0.5 ml min^−1^ with a gradient of acetonitrile from 85% to 100% over 10 min, followed by a column wash phase and re‐equilibrium step at 1 ml min^−1^ and detection at 254 nm. ABT for calibration purposes was synthesized according to Ingram et al. (2007).

### 2.2 Fabrication of microreactor and micromixer

All components were designed using Solidworks® (Dassault Systems, France). The microreactor and the micromixer were comprised of two rigid poly(methylmethacrylate) (PMMA) layers; 1.5 mm thick for the microreactor layers, and 2 mm thick for the micromixer layers. The channels and cut‐outs were fabricated using a CO_2_ laser marking head (Epilog Laser, UK) and the layers were thermally bonded by clamping together at 400 cNm^2^ for 45 min at 110°C. Standard connection fittings (P‐221, Upchurch Scientific) were used to attach polytetrafluoroethylene tubing (PTFE, ID 0.75 mm, VWR International Ltd, UK).

### 2.3 Biocatalyst preparation

#### 2.3.1 Strains and plasmids

Transketolase (WT‐TK from *Escherichia coli* BL21gold DE3 containing the plasmid pQR791) (Martinez‐Torres, Aucamp, George, & Dalby, 2007) and ω‐transaminase producing strains (CV2025 from *E. coli* BL21gold DE3 containing the plasmid pQR801) (Kaulmann, Smithies, Smith, Hailes, & Ward, 2007) were produced in‐house and stored at ‐80°C in LB broth containing 50% (v/v) glycerol.

#### 2.3.2 Culture conditions and enzyme expression

##### Transketolase

Overnight cultures were prepared in 10 g L^−1^ LB broth supplemented with 150 µg ml^−1^ ampicillin and 10 g L^−1^ glycerol. Cells were sub‐cultured using 1% (v/v) inoculum in 2 L shake flasks containing 500 ml of the same supplemented LB broth at 37°C and 250 rpm until the bacterial growth reached stationary phase. Cells were harvested by centrifugation at 8,000 rpm for 20 min at 4°C.

##### Transaminase

Overnight cultures were prepared in 10 g L^−1^ LB broth supplemented with 30 µg ml^−1^ kanamycin and 10 g L^−1^ glycerol. Cells were sub‐cultured using 1% (v/v) inoculum in 2 L shake flasks containing 500 ml of the same supplemented LB broth at 37°C and 250 rpm. Transaminase expression was induced with 1 mM of isopropyl β‐D‐1‐thiogalactopyranoside (IPTG, Calbiochem) when growing in early exponential phase, and temperature was reduced to 30°C. PLP was added to a final concentration of 400 µM at least 15 min before harvesting. Cells were harvested by centrifugation at 8,000 rpm for 20 min at 4°C, 5 hr after induction.

For experiments performed at pH 9, the cells were grown in Terrific Broth 46.7 g L^−1^, induced at OD 0.3 with 0.1 mM IPTG and shaken overnight at 200 rpm. No PLP was added before harvesting. Cells were harvested by centrifugation at 8,000 rpm for 20 min at 4°C.

##### Lysate preparation

The cell pellets were resuspended in either 50 mM Tris–HCl, pH 7.0 or pH 9, according to the different pH experiments, and sonicated on ice (Soniprep 150, MSE Sanyo, Japan). The suspension was centrifuged at 13,000 rpm at 4°C for 20 min and the supernatant removed from the cell debris. The clarified cell lysates were stored at −20°C until use.

### 2.4 Biotransformations

#### 2.4.1 Transketolase batch reactions

Reactions were performed in 10 ml vials filled with 7 ml of reaction media and mixed by magnetic stirring. Substrate concentrations were 50 mM HPA and 50 mM GA in the vial, and TK concentrations ranged from 0.63 to 2.85 U ml^−1^. The concentrations of ThDP and MgCl_2_ were 2.4 and 9.8 mM for the lowest enzyme concentration. The cofactor concentrations were increased by a factor 1.25 for each increase in enzyme concentration, so that the cofactor concentrations were 9.395 mM ThDP and 38.28 mM MgCl_2_ for the highest enzyme concentration. All solutions were prepared in 50 mM Tris–HCl pH 7.0. Prior to reaction, TK was incubated with the cofactors for 30 min. Aliquots of 50 μl were taken at various time intervals and quenched with 450 μl 0.1% (v/v) aqueous TFA, centrifuged (5,000 rpm, 5 min) and the supernatant analyzed by HPLC as described above.

#### 2.4.2 Transketolase flow reactions

Two syringes, one containing transketolase (TK ranging from 2.00 to 8.07 U ml^−1^, with cofactors ThDP 4.8 mM and MgCl_2_ 19.6 mM), the other containing the substrate solutions (100 mM HPA and GA), were connected to the microreactor. Both solutions were pumped (KDS210, KD Scientific, Holliston) at the same flow rate, which varied between 1 and 60 μl min^−1^ (i.e., 2 and 120 μl min^−1^ total flow rate in the reaction channel), depending on the desired residence time. To ensure the measurements were performed when the microreactor was in steady state, samples were taken after three (mean) residence times. Samples were quenched with 0.1% (v/v) TFA.

#### 2.4.3 Transaminase batch reactions

Reactions were performed in 1.5 ml Eppendorf tubes containing 25 mM ERY, purchased from Sigma–Aldrich, UK, 10 mM MBA, 1 mM PLP, and TAm in the range of 1.90 and 5.24 U ml^−1^. Solutions were thoroughly mixed with a pipette and allowed to react at room temperature (20°C). Samples were removed at the required time intervals, quenched with 0.1% (v/v) TFA, centrifuged (5,000 rpm, 5 min) and the supernatant analyzed by HPLC.

#### 2.4.4 Transketolase‐transaminase cascade flow reactions

**Figure 1:**
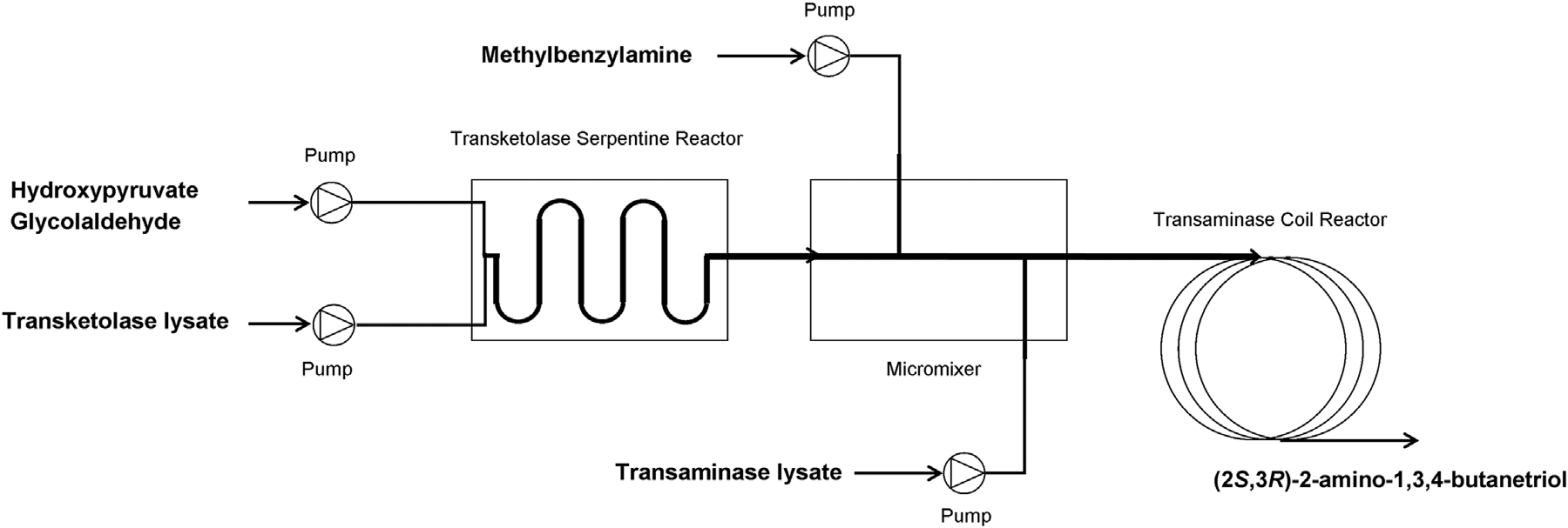
Scheme of the microfluidic setup for the two‐step transketolase‐transaminase catalyzed synthesis of (2*S*,3*R*)‐2‐amino‐1,3,4‐butanetriol (ABT). Hydroxypyruvate and glycolaldehyde were brought together in a serpentine microreactor with the transketolase (TK) lysate. The product of the reaction, L‐erythrulose, was fed into a micromixer where it was mixed with (S)‐α‐methylbenzyl‐amine and the second enzyme lysate, containing transaminase (TAm). The TAm reaction took place in a coil reactor where (2*S*,3*R*)‐2‐amino‐1,3,4‐butanetriol was produced

A fluidic path was created by connecting the transketolase microreactor, a micromixer and a transaminase coil reactor (PTFE coil, ID 0.75 mm and 6.43 m long), with Upchurch® connectors and fittings (P‐221, Upchurch Scientific). Syringe pumps (KDS210, KD Scientific, Holliston) which were connected to the reactors at different points in the fluidic path (Figure 1). Transketolase reactions were carried out under identical conditions as previously described (section 2.6.2) with an activity of 3.25 U ml^−1^. For the transaminase reaction, one syringe containing 40 mM MBA (pH 10, initial pH), and a second containing 27.0 U ml^−1^ TAm lysate and 2 mM and PLP respectively at pH 9, were connected to the micromixer as shown in Figure 1 (final concentrations of 10 mM MBA and 10.8 U ml^−1^ TAm were thus obtained). The flow rate for the TAm syringe was varied from 2 to 40 μl min^−1^. All other inlet flows were set at half the TAm flow rate. Samples were taken after three (mean) residence times, quenched with 0.1% (v/v) TFA and analyzed by HPLC as described above. To compare batch and continuous reactions the residence times were normalized according to Marques, Fernandes, Cabral, Žnidaršič‐Plazl, and Plazl (2012).

## 3 RESULTS AND DISCUSSION

### 3.1 Reactors for the transketolase‐transaminase cascade

**Figure 2:**
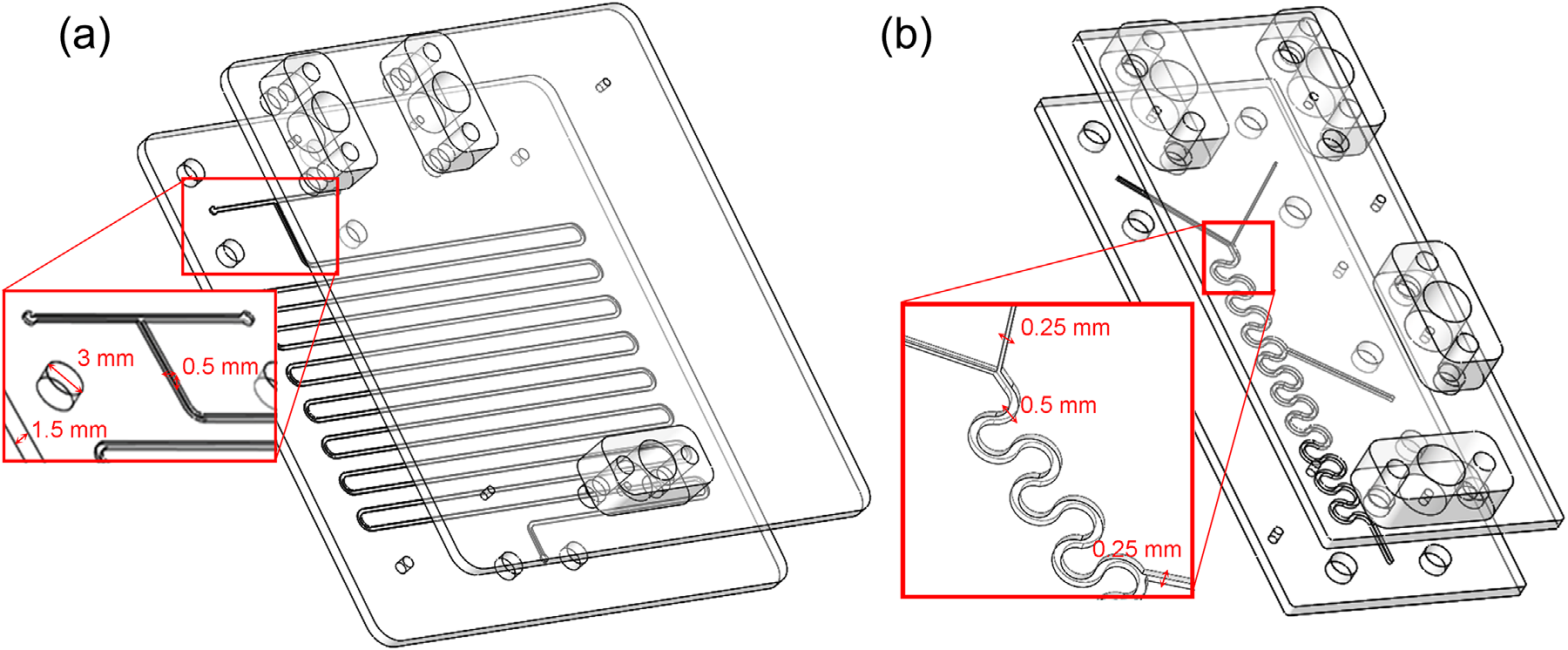
(a) Exploded view of the transketolase microreactor made out two layers of poly(methylmethacrylate) (PMMA), a reaction layer and a cover layer; detail view shows the geometry of the inlet channels and the T‐junction with the reaction channel; six large bores in each layer hold the connector bars for the fluidic interconnection. (b) Exploded view of the micromixing device made out two layers of PMMA, a layer with the mixing structure and a cover layer. Detail view shows the geometry of the inlets; the Y‐junction at the top of the meandering channel to add the co‐substrate for the transaminase‐catalyzed reaction, and the inlet in the middle of the channel to introduce the transaminase

The transketolase (TK) reaction was performed in a microreactor with a serpentine reaction channel (500 µm wide, 500 µm deep) and a working volume of 240 µl. The TK enzyme and the two substrates were brought together in the reaction channel with a T‐junction (Figure 2a). Downstream of the outlet of the TK microreactor, the co‐substrate for the transaminase reaction, and the transaminase (TAm) were added to the product stream of the TK microreactor using a micromixer. The device contained a Y‐junction followed by a meandering channel (500 µm wide, 450 µm deep, and internal volume of 200 µl) to enhance the mixing (Figure 2b). At the point of confluence of the Y‐junction, the co‐substrate was mixed with the product from the TK reaction. The TAm was introduced in a second inlet further downstream of the Y‐junction to ensure that the co‐substrate of the TAm reaction had mixed with the product from the TK reaction before the TAm enzyme was added. The devices were connected as described in section 2.4.4 to form a microfluidic flow path for the transketolase‐transaminase reaction cascade using the transaminase coil reactor to provide sufficiently long residence times. The fluidic connector bars were fabricated according to previously reported designs (Gruber, Marques, Sulzer et al., 2017; Lawrence, O'Sullivan, Lye, Wohlgemuth, & Szita, 2013).

### 3.2 Optimization of the transketolase reaction in the microreactor

In a previous study of the production of L‐erythrulose (ERY) from hydroxypyruvate (HPA) and glycolaldehyde (GA), full conversion of 50 mM of HPA and GA was achieved in approximately 2 hr using 2.28 mg ml^−1^ of total lysate protein (O'Sullivan et al., 2012). In the present work, transketolase concentrations were varied between 1.95 and 7.66 mg ml^−1^ (corresponding to 1–4 U ml^−1^, respectively) while substrate concentrations were maintained at 50 mM, in order to explore the maximal volumetric productivity achievable with the reactor. The time to achieve full conversion was expected to reduce as the TK concentration increased. The conversion time would decrease either until eventually a plateau would be reached where the enzyme is in excess and the reaction becomes mass transfer limited; or where an upper limit of the enzyme concentration would be reached, imposed by the solubility of TK in the reaction medium.

The enzyme concentrations and the corresponding observed reaction rates for this optimization study were summarized in Table 1. A maximum initial rate of 1.19 × 10^−2^ mmol ml^−1^ min^−1^ was achieved for a TK activity of 0.77 U per reactor volume; a higher activity per reactor volume did not further increase the reaction rate. Therefore, this set a practical upper limit to the enzyme concentration in the optimization studies, indicating that the maximum volumetric productivity was attained. The complete reaction profiles for the six conditions used are presented in Figure S1, Supporting Information.

**Table 1:** Transketolase reaction conditions and corresponding times of conversion

Enzyme concentration (U ml^−1^)	Protein concentration (mg ml^−1^ in reactor)	Enzyme concentration (U per reactor volume)	Initial rate (mM min^−1^)	Time for complete conversion (min)
1.00	0.47	0.24	2.22	45
1.60	0.75	0.38	3.99	30
2.00	0.94	0.48	5.96	15
2.58	1.18	0.62	8.14	12
3.25	1.47	0.77	11.93	8
4.04	1.84	0.97	11.87	8

**Figure 3:**
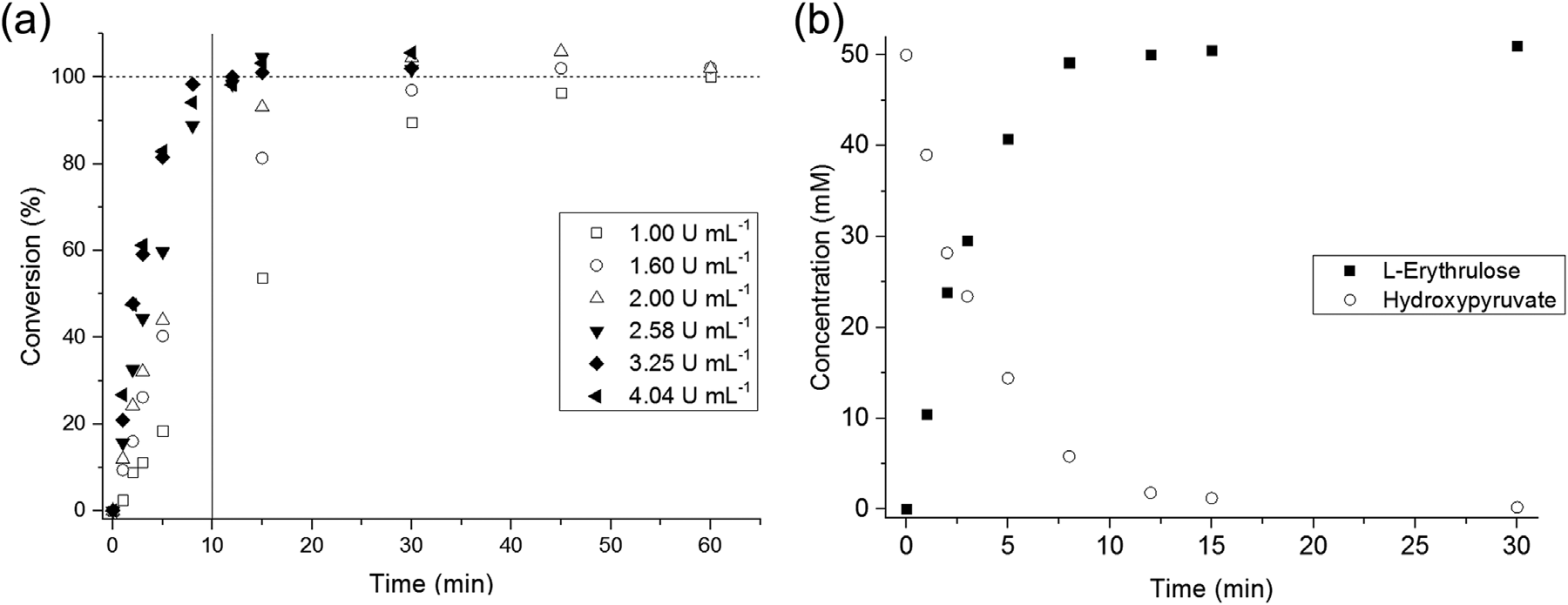
(a) Transketolase reaction profile for the production of L‐erythrulose (ERY) at various enzyme activities. A total of 100% conversion corresponded to the production of 50 mM of ERY (dashed line). (b) Transketolase reaction profile at 3.25 U ml^−1^ (i.e., 0.77 U per reactor volume) showing the production of ERY and consumption of hydroxypyruvate (HPA). All reactions were performed at 20°C with initial concentrations of HPA and glycolaldehyde (GA) of 50 mM. Experiments were performed in duplicate (*n* = 2) with one standard error below 5%

Full conversions were achieved for all conditions, but the residence times required were significantly reduced by the increased enzyme concentrations (Figure 3). For an increase in TK activity from 0.24 U to 0.77 U (and 0.97 U) the conversion time reduced from 45 min to just 8 min. Thus we achieved a >5‐fold decrease of the conversion time between the conditions applied in this reactor, and a 15‐fold decrease compared to our previous results (O'Sullivan et al., 2012). The quantification of ERY was performed using calibration curves derived from standard solutions of purified ERY, which explained conversions seemingly higher than 100%.

The experimental results obtained in the microreactor were compared with reactions in magnetically stirred vessels for identical substrate and enzyme concentrations. As in the case of the flow reactions, the initial reaction rate increased with higher concentrations of transketolase (data not shown).

### 3.3 Optimization of the transaminase reaction

The transaminase‐catalyzed reaction of ERY with MBA to (2*S*,3*R*)‐2‐amino‐1,3,4‐butanetriol (ABT) and acetophenone (AP) has a much lower reaction rate constant than the TK‐catalyzed reaction of GA with HPA to ERY and CO_2_. According to (Matosevic, Lye, & Baganz, 2011), the initial rate of TK is fourfold higher than that of the initial rate of TAm, thus more than three orders of magnitude lower than the reported reaction rate for transketolase. To find the maximal productivity, batch reactions using 25 mM ERY and 10 mM MBA were performed with increasing concentrations of biocatalyst. The excess of ERY, used throughout this present work, was chosen to overcome the unfavorable reaction equilibrium (Villegas‐Torres et al., 2015); though we chose a much lower excess of ERY than previously reported in order to avoid a large excess of ERY which would render potential downstream purification steps more challenging. The concentration of the amine donor (MBA) was fixed at 10 mM due to the inhibitory effects on the enzyme for higher concentrations (Halim et al., 2014). The highest transaminase concentration tested in batch was 5.24 U ml^−1^ (data not shown).

**Figure 4:**
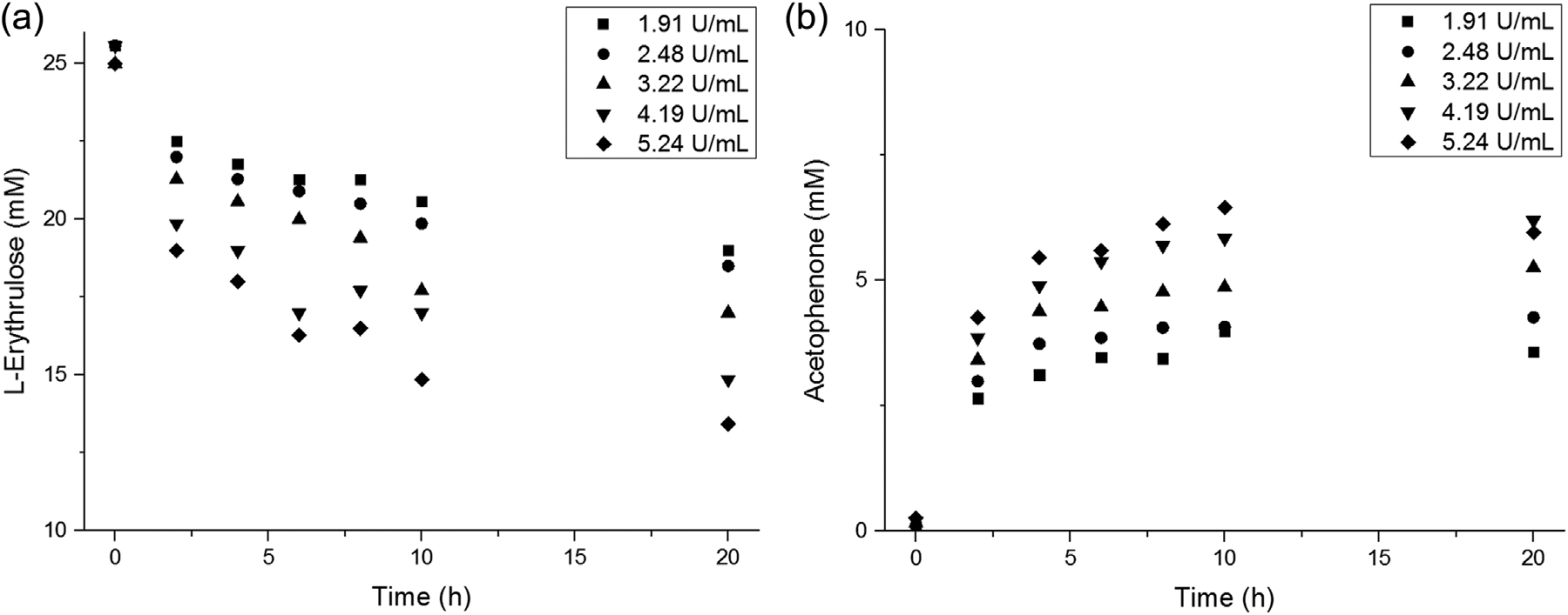
Transaminase reaction profiles of the production (2*S*,3*R*)‐2‐amino‐1,3,4‐butanetriol (ABT) showing how the consumption of L‐erythrulose and the production of by‐product acetophenone (AP) varied with increasing enzyme concentrations. All reactions were performed at 20°C with initial concentrations of 25 mM L‐erythrulose (ERY) and 10 mM (S)‐α‐methylbenzylamine (MBA). Experiments were performed in duplicate (*n* = 2) and the resulting standard error was below 5% (not plotted)

Increasing the transaminase concentration led to higher conversion yields within shorter residence times (Figure 4). Full conversion was achieved after 20 hr with the two highest TAm concentrations, 4.2 and 5.24 U ml^−1^. After 10 hr, with a TAm concentration of 5.24 U ml^−1^ the conversion was approximately 90%. Unlike in the transketolase optimization study, for this enzyme, we did not observe a plateau of initial reaction rate when comparing the reaction rates for enzyme concentrations between 1.91 and 5.24 U ml^−1^.

### 3.4 Transketolase cofactor optimization for coupled TK‐TAm reaction

**Figure 5:**
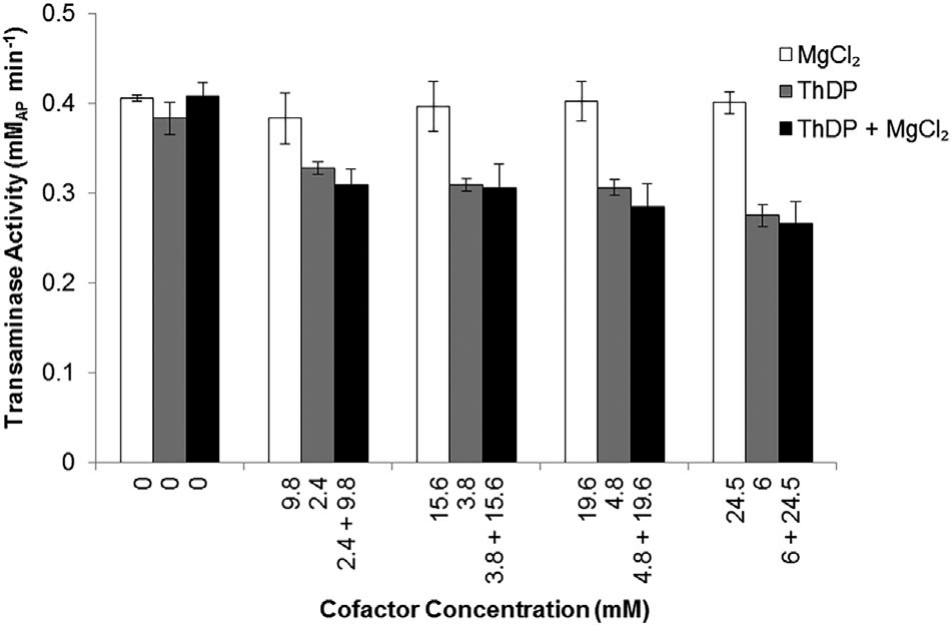
Effect of transketolase cofactors, thiamine pyrophosphate (ThDP) and MgCl_2_, on the transaminase activity. Activity was determined through photometric detection of acetophenone formation at 280 nm and given in mM_AP_ min^−1^. The reactions were performed at 20°C with initial concentrations of 10 mM sodium pyruvate and 10 mM (S)‐α‐methylbenzylamine (MBA). Experiments were performed in quadruplicates (*n* = 4) and error bars represented ± 1 standard deviation about the mean

Initially, when we performed the TK‐catalyzed and TAm‐catalyzed reactions in sequence, we did not detect any ABT or AP formation (data not shown). As a result, we tested all compounds of the TK reaction for any inhibitory effects on the transaminase, and we discovered that the activity of transaminase was significantly affected by the concentration of the thiamine pyrophosphate (ThDP) cofactor, while the concentration of the magnesium chloride had no effect (Figure 5). Indeed, the transaminase activity decreased by approximately 25% when adding 2.4 mM of ThDP (compared with 0 mM ThDP). For higher concentrations of ThDP, the activity decreased only marginally. We therefore tested a value of ThDP smaller than 2.4 mM for the TK reaction and we found that the reaction proceeded at similar rates (as found during the TK optimization study, section 3.2) with a ThDP concentration of 1.2 mM. For this concentration of ThDP, which was half the concentration used in the TK optimization study for the smallest enzyme concentration (section 3.2), we still obtained conversion times of approximately 10 min for the TK reaction.

### 3.5 Transketolase‐transaminase cascade reactions

Having optimized the co‐factor concentrations, we then evaluated the performance of the microfluidic reaction cascade (Figure 1). Based on the results obtained in section 3.4, we chose to investigate the TAm reaction with (mean) residence times between 30 min and 10 hr. For a coil reactor with a volume of 3 ml, flow rates between 5 and 100 μl min^−1^ were required. Higher flow rates would have required a longer coil reactor to achieve sufficiently long residence times, and lower flow rates would be less reliable to execute (for example, a flow rate of 5 μl min^−1^ for the TAm reaction step meant a flow rate of only 1 μl min^−1^ for the co‐substrate MBA). As a result of this choice of flow rates for the TAm reaction step, the TK reactions were therefore performed with flow rates between 2 and 40 μl min^−1^, that is, residence times between 120 and 6 min, respectively. Additionally, we used the compartmentalization afforded by the two microfluidic reactors to perform each reaction at a different individual pH. The results of section 3.4 had shown that performing the TAm reaction at pH 7 led to long reaction times. Therefore, the TK reaction was performed at pH 7, and then the solution entering the coil reactor was adjusted to a pH 9 for the TAm reaction step (Schell et al., 2009).

**Figure 6:**
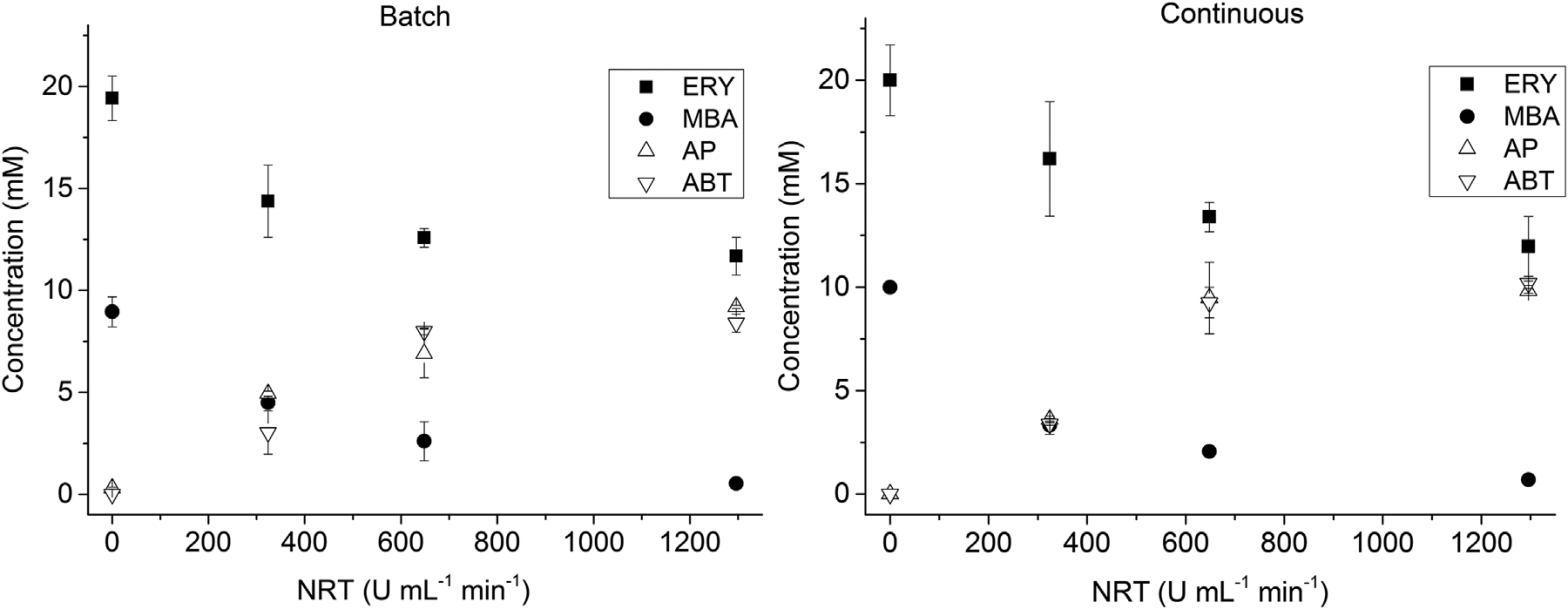
Reaction profiles for the coupled transketolase‐transaminase reaction a sequentially added batch (left) and in a microfluidic reaction cascade (right) for residence times up to 2 hr. The reactions were performed at 20°C with an initial concentration of 50 mM of GA and HPA for the TK reaction, and with an initial substrate concentrations in flow of 20 mM L‐erythrulose (ERY), 10 mM (S)‐α‐methylbenzylamine (MBA) with transketolase and transaminase enzyme activity of 3.25 U ml^−1^ and 10.8 U ml^−1^, respectively. The TK reaction was performed at a starting pH of pH 7, while the TAm reaction was performed at pH 9. The TK reaction was not the rate‐limiting step in the cascade system and therefore neglected. Reactions were performed in triplicates (*n* = 3), error bars representing one standard deviation. To compare batch and continuous reactions the residence times were normalized according to Marques et al. (2012).

As can be seen from Figure 6, 10 mM of ABT were obtained after 2 hr both in the continuous microfluidic system and in the batch system. Also, within the same time, we observed a full conversion of MBA to AP. Achieving full conversion in 2 hr meant a 10‐fold reduction of the reaction time from the 20 hr in Figure 4. This dramatic improvement in space‐time yield was primarily due to the optimization of the reaction conditions, such as the pH for the TAm reaction, a higher activity of the enzyme and the reduced concentration of the co‐factors for the TK reaction. Furthermore, the ERY used in the batch experiments (Figure 4) was from a different origin and produced by a fermentation process. It was a hygroscopic solution with a water content of 14.2% and showed small amounts of impurities (data not shown). These impurities were not present in the ERY produced in situ biocatalytically (with the TK), which shows the advantage of producing an intermediate in a biocatalytic reaction sequence.

The ABT concentration obtained in our microfluidic reactor cascade is comparable with or higher than other transaminase‐catalyzed reaction steps in flow systems. A final 5 mM ABT concentration was achieved in a capillary packed bed reactor with purified immobilized enzymes (1.1 ± 0.15 and 0.15 ± 0.006 mg ml^−1^ of TAm and TK used, respectively) (Abdul Halim et al., 2013). Using an initial substrate concentration of 60 mM HPA and GA, and 6 mM MBA (i.e., a 10:1 ratio of ERY:MBA), the final concentration of ABT (83% conversion) was reached in 20 min. In contrast, in our system we only required a 2:1 ratio, which significantly facilitates recovery of the final compound. Matosevic et al. (2011) immobilized both the TK and TAm onto AB‐NTA derivatized microcapillaries in order to produce 0.3 mM of ABT in 40 min using 10 mM MBA, HPA, and GA (i.e., an equimolar ratio of ERY:MBA), though ABT was not directly measured. Full conversion was not shown, but could be estimated to be achievable after 45 hr, which is more than an order of magnitude larger than the 2 hr to full conversion reported here.

ABT has traditionally been produced in one‐pot batch systems using the TK‐TAm enzymatic reactions, yet full conversion was not demonstrated. Ingram et al. (2007) obtained a final concentration of 5 mM ABT (corresponding to a 21% mol/mol yield conversion of L‐erythrulose to ABT) over 100 hr of reaction time. However, the corresponding conversion of MBA to AP and the depletion of ERY were each clearly below 100%. More recently, Villegas‐Torres et al. (2015) showed a conversion limitation of ABT at 2.5 mM after 50 hr using L‐serine instead of MBA as a co‐substrate for the TAm reaction. Side reactions limited the attainable conversion, which illustrates a disadvantage of one‐pot systems: the difficulty of achieving optimal reaction conditions for both enzymes. To successfully achieve full conversion of the coupled TK‐TAm reaction, it is mandatory to obtain full conversion of the substrates of the first enzymatic step; GA and HPA are preferable substrates for the reaction catalyzed by the CV2025 transaminase. The transamination of HPA can for example lead to the formation of serine (Rios‐Solis et al., 2011; Villegas‐Torres et al., 2015). Full conversion in the TAm reaction step was achieved by Halim et al. (2014). However, they used purified ERY instead of forming this substrate with a coupled TK‐catalyzed reaction step upstream. And additionally, the TAm reaction step either required a secondary reaction to shift the reaction equilibrium, or the implementation of in situ product removal strategies (ISPR).

**Figure 7:**
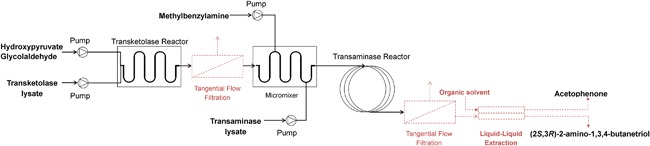
Conceptual setup of an ideal cascading reaction system for the continuous production of (2*S*,3*R*)‐2‐amino‐1,3,4‐butanetriol (ABT) using free enzymes. The transketolase and transaminase reactions are compartmentalized and the enzymes are recovered through tangential flow filtration units (Lawrence et al., 2013). ABT is separated from co‐substrate acetophenone using a liquid–liquid extraction device (Chiang, Dimov, & Szita et al., 2016)

A number of routes could be pursued to further improve ABT production; by using purified enzyme the volumetric activity per unit volume could be increased; by using time‐controlled additions of MBA using a microfluidic side‐entry reactor (Gruber, Marques, Sulzer et al., 2017; Lawrence et al., 2013) the space‐time yield could be increased; by adding optical sensors and control strategies to maintain optimal reaction conditions (Gruber, Marques, Szita, & Mayr, 2017); by increasing the ERY to MBA ratio the equilibrium could be shifted further toward the product side (Scheme 1) (though at the expense of recovery efforts); or finally by implementing ISPR strategies (Gruber, Marques, O'Sullivan et al., 2017). For the latter, the use of a chromatography or a liquid‐liquid extraction unit operation could be envisaged as an ISPR step. As an example of such a system (Figure 7), a filtration step between the TK reactor and the micromixer could be added to remove and recycle the TK. A similar filtration step could also be added after the TAm coil reactor to recycle this enzyme. The permeate from this filtration step could be fed into a liquid‐liquid extraction unit to facilitate recovery of the final compound. A fully assembled synthesis and purification system as shown in Figure 7 could then become a first step toward an automated continuous‐flow production of ABT.

## 4 CONCLUSIONS

Using continuous‐flow microreactors, we have optimized the two‐step enzymatic synthesis of the chiral amino‐triol (2*S*,3*R*)‐2‐amino‐1,3,4‐butanetriol (ABT), an important building block for statins and intermediate in the synthesis of detoxinine. Our results have shown the use of cascading free enzymes in microfluidic devices for the first time, with significant improvement in conversion yields over free enzymes in one‐pot systems (Ingram et al., 2007) and over immobilized enzymes in microfluidic systems (Abdul Halim et al., 2013; Matosevic et al., 2011) The transketolase (TK)‐transaminase (TAm) reaction cascade was optimized according to Gruber, Marques, O'Sullivan et al. (2017) to overcome key issues, such as substrate and cofactor inhibition, and we have obtained for the first time full conversion in both continuous‐flow microreactors and batch systems.

We have successfully reduced the conversion time of the first reaction step, the hydroxypyruvate (HPA) and glycolaldehyde (GA) conversion into L‐erythrulose (ERY), from 2 hr (O'Sullivan et al., 2012) to less than 10 min using a TK enzyme activity of 3.25 U ml^−1^. When coupled with the TAm reaction, using (S)‐α‐methylbenzylamine (MBA) as a co‐substrate and increasing the volumetric activity of the TAm up to 10.8 U ml^−1^, a final yield of 100% ABT and a full conversion of MBA in 2 hr was achieved. Furthermore, the spatial confinement of the two enzymatic reactions enabled us to detect a previously unreported inhibition effect of thiamine diphosphate (ThDP), the cofactor of TK (the first enzyme in the cascade), on the rate of the second enzyme, TAm. This discovery could in principle have been obtained with a suitable configuration of (mini)batch reactors, though at the expense of not arriving at a continuous synthesis of ABT.

The modular microfluidic system offers flexibility for further engineering adaptations and improvements to increase the productivity of biocatalytic systems. These improvements can either relate to reactor engineering, for example by redesigning reactors to allow the additions of substrates at different points in the reaction in order to avoid enzyme deactivation due to substrate inhibition (Gruber, Marques, Sulzer et al., 2017; Lawrence et al., 2013), or relate to process monitoring and control strategies (Gruber, Marques, Szita et al., 2017), or relate to the implementation of in situ product removal strategies to drive the reaction equilibrium toward the formation of ABT (Gruber, Marques, O'Sullivan et al., 2017; Halim et al., 2014).

Multi‐step enzymatic syntheses are of increasing interest as straightforward and sustainable routes from simple starting materials to chiral amino‐alcohols (Lorillière et al., 2017; Sehl, Maugeri, & Rother, 2015b; Villegas‐Torres et al., 2015). This work has established modular microfluidic devices as a versatile and powerful tool for biocatalytic process optimization and intensification and as a platform for the investigation of such cascade reactions using enzymes in their free form without the need for immobilization.

## CONFLICTS OF INTEREST

The authors declare no financial or commercial conflict of interest.

## Supporting information

Additional Supporting Information may be found online in the supporting information tab for this article.

**Figure S1**. Transketolase reaction profile of the production L‐erythrulose at various enzyme activities: A – 1.00 U ml^−1^, B – 1.60 U ml^−1^, C – 2.00 U ml^−1^, D – 2.58 U ml^−1^, E – 3.22 U ml^−1^, F – 4.04 U ml^−1^.
