# Supplementary material for: Enzymatic synthesis of chiral amino‐alcohols by coupling transketolase and transaminase‐catalyzed reactions in a cascading continuous‐flow microreactor system
Source: Biotechnol Bioeng. 2017 Nov 9;115(3):586–96. doi: 10.1002/bit.26470 (PMC5813273; doi:10.1002/bit.26470)
Supplement: Supplementary file 1 — Figure S1. Transketolase reaction profile of the production L‐erythrulose at various enzyme activities: A – 1.00 U ml−1, B – 1.60 U ml−1, C – 2.00 U ml−1, D – 2.58 U ml−1, E – 3.22 U ml−1, F – 4.04 U ml−1. [file BIT-115-586-s001.docx]

**Supporting Material** – Gruber et al., Enzymatic Synthesis of Chiral Amino-Alcohols by Coupling Transketolase and Transaminase-Catalyzed Reactions in a Cascading Continuous-Flow Microreactor System

1. Transketolase optimisation in flow


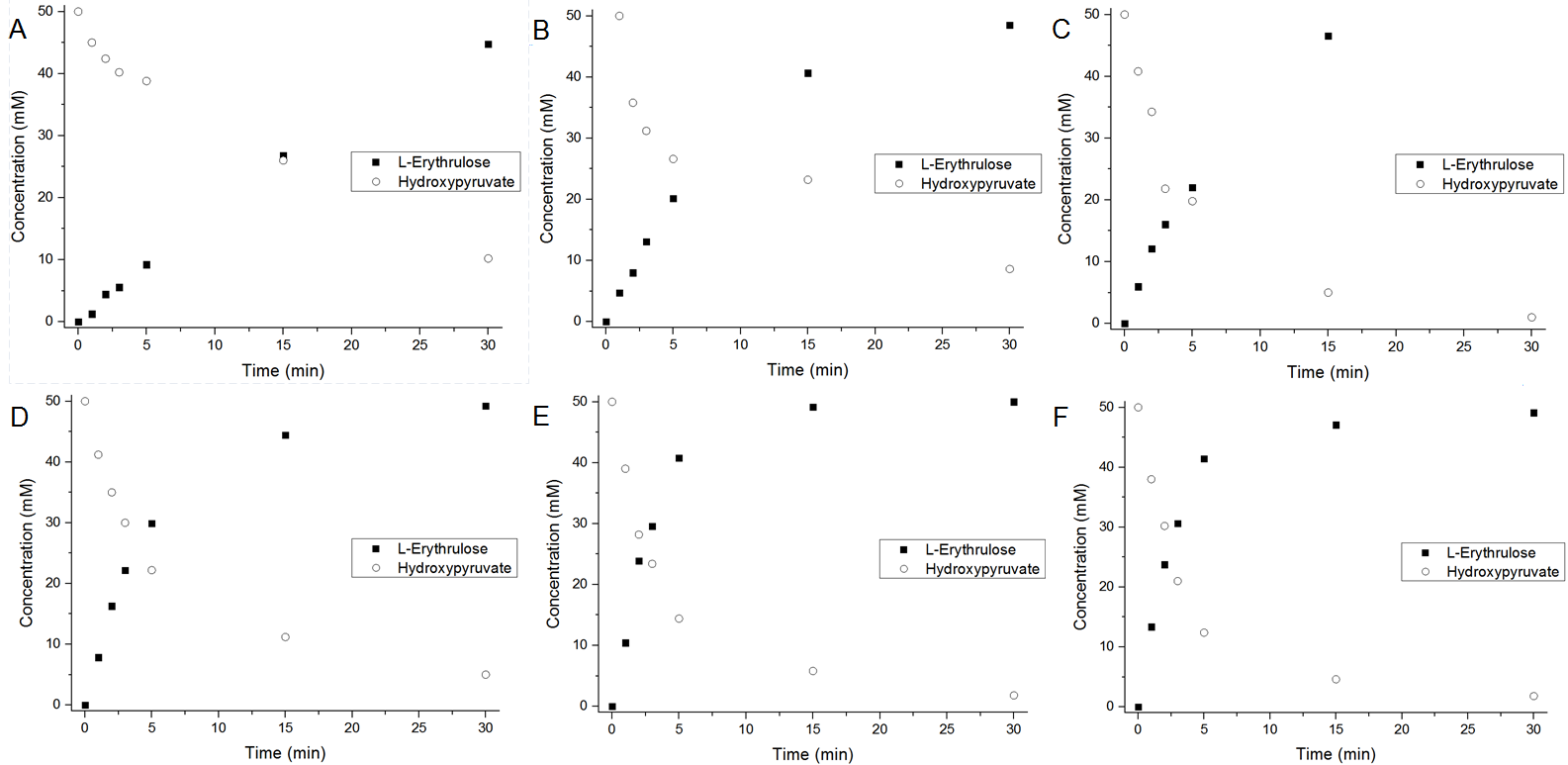


Supporting Figure 1: Transketolase reaction profile of the production L- erythrulose at various enzyme activities: A – 1.00 U∙mL^-1^, B – 1.60 U∙mL^-1^, C- 2.00 U∙mL^-1^, D 2.58 U∙mL^-1^, E- 3.22 U∙mL^-1^, F – 4.04 U∙mL^-1^
